# Supplementary material for: Global-scale drivers of crop visitor diversity and the historical development of agriculture
Source: Proc Biol Sci. 2019 Nov 20;286(1915):20192096. doi: 10.1098/rspb.2019.2096 (PMC6892048; doi:10.1098/rspb.2019.2096)
Supplement: Supplementary material for “Global scale drivers of crop visitor diversity and the historical development of agriculture” ; Data S1 [file rspb20192096supp1.docx]

**Supplementary material for**

**Global scale drivers of crop visitor diversity and the historical development of agriculture**

DOI: 10.1098/rspb.2019.2096

Julian Brown and Saul A. Cunningham

Correspondence to: julian.macpherson.brown@gmail.com

Table S1.

List of terms used in regression modelling

| TERM | DEFINITION |
| --- | --- |
| Number of bee genera | Count of bee genera observed visiting one crop across all locations in one study (response variable) |
| Latitude | Degrees of latitude for the location of the study (or centroid for multiple locations) |
| Number of locations | Number of locations (i.e. survey sites assumed by authors to be independent spatial replicates) in which flower visitors were observed on the focal crop in the study |
| Realm of study | The biogeographic realm in which the study was conducted (5 levels) |
| Crop origin | Two levels, 0 = observations made outside realm where crop originated, 1 = observations made in the realm where crop originated (note where crop origin could not be clearly located in a single realm it was located in the most likely set of realms) |
| Family origin | Two levels, 0 = observations made outside realm where crop’s family originated, 1 = observations made in the realm where crop’s family originated (note where family origin could not be clearly located in a single realm it was located in the most likely set of realms) |
| Crop species | The crop for which bees were observed, 27 levels. Crop species was included as a random effect to account for intrinsic differences between species. |

**Table S2.**

Change in AIC relative to best model (∆ AIC) and Aikaike weights (*w*) for top ten models. Tick mark indicates presence of predictor variable in the model.

| **Latitude** | **Locations** | **Crop origin** | **Family origin** | **Biogeographic Realm** | **∆ AIC** | ***w*** |
| --- | --- | --- | --- | --- | --- | --- |
| ✓ | ✓ | ✓ | ✓ | ✓ | 0 | 77 |
|  | ✓ | ✓ | ✓ | ✓ | 3 | 21 |
| ✓ | ✓ | ✓ |  | ✓ | 8 | 1 |
| ✓ | ✓ |  | ✓ | ✓ | 10 | 0 |
|  | ✓ | ✓ |  | ✓ | 12 | 0 |
|  | ✓ |  | ✓ | ✓ | 14 | 0 |
| ✓ | ✓ |  |  | ✓ | 18 | 0 |
|  | ✓ |  |  | ✓ | 24 | 0 |
| ✓ | ✓ | ✓ | ✓ |  | 37 | 0 |
|  | ✓ | ✓ | ✓ |  | 39 | 0 |

**Table S3**: Biogeographic realm of family and crop origin, and number of studies (with reference numbers from Data S1) describing visitors for each crop in each realm.

| **Family** | **Family origin** | **Crop** | **Crop origin** | **Old World** | | | **New World** | |
| --- | --- | --- | --- | --- | --- | --- | --- | --- |
|  |  |  |  | **Palearctic** | **Afrotropics** | **Indomalaya** | **Nearctic** | **Neotropic** |
| Asteraceae | Neotropic | *Carthamus tinctorius* | Palearctic | 2 (74, 316) | NIL | 6 (55, 195, 196, 214, 239, 269) | 1 (8) | NIL |
| Asteraceae | Neotropic | *Helianthus annuus* | Nearctic | 14 (29, 34, 36, 46, 58, 81, 84, 103, 104, 130, 209, 229, 250, 315) | 2 (62, 138) | 19 (8, 11, 18, 26, 40, 119, 132, 155, 156, 173, 213, 215, 238, 239, 243, 277, 278, 285, 288) | 11 (47, 64, 65, 80, 116, 144, 169, 218, 231, 261, 296) | 6 (56, 70, 126, 162, 187, 297) |
| Cucurbitaceae | Indomalaya | *Citrullus lanatus* | Afrotropic or Palearctic | 3 (109, 228, 289) | 1 (201) | 3 (19, 44, 203) | 6 (53, 59, 60, 112, 121, 151) | 4 (168, 179, 222, 284) |
| Cucurbitaceae | Indomalaya | *Cucumis melo* | Indomalaya | 1 (251) | 1 (150) | 6 (19, 44, 117, 157, 194, 247) | 1 (307) | 4 (142, 143, 179, 280) |
| Cucurbitaceae | Indomalaya | *Cucumis sativas* | Indomalaya | 1 (45) | NIL | 8 (32, 41, 86, 108, 120, 189, 270, 295) | 6 (35, 96, 139, 160, 161, 281) | 1 (179) |
| Cucurbitaceae | Indomalaya | *Cucurbita moschata* | Neotropic | 1 (223) | 1 (14) | 1 (124) | 2 (122, 133) | 5 (152, 179, 222, 267, 314) |
| Cucurbitaceae | Indomalaya | *Cucurbita pepo* | Nearctic or Neotropic | 3 (12, 85, 149) | NIL | 6 (20, 114, 117, 244, 254, 309) | 2 (25, 298) | 4 (28, 69, 93, 152) |
| Fabaceae | Afrotropic or Palearctic | *Cajanus cajan* | Indomalaya | NIL | 4 (98, 207, 211, 217) | 15 (10, 15, 52, 118, 165, 186, 232, 242, 245, 271, 272, 276, 295, 305, 310) | NIL | 1 (30) |
| Fabaceae | Afrotropic or Palearctic | *Glycine* | Palearctic | 1 (312) | NIL | NIL | 1 (256) | 3 (66, 180, 183) |
| Fabaceae | Afrotropic or Palearctic | *Phaseolus coccineus* | Nearctic or Neotropic | 5 (48, 105, 140, 232, 308) | 2 (99, 216) | NIL | 1 (298) | NIL |
| Fabaceae | Afrotropic or Palearctic | *Phaseolus vulgaris* | Nearctic or Neotropic | NIL | 4 (88, 137, 145, 176) | NIL | 1 (298) | 3 (123, 125, 259) |
| Fabaceae | Afrotropic or Palearctic | *Vigna unguiculata* | Afrotropic | NIL | 5 (27, 95, 97, 129, 287) | 3 (219, 237, 295) | NIL | 2 (37, 221) |
| Malvaceae | Neotropic | *Gossypium hirsutum* | Neotropic | 2 (33, 266) | 2 (89, 286) | 6 (1, 83, 193, 230, 275, 301) | 2 (71, 158) | 5 (70, 90, 136, 226, 282) |
| Malvaceae | Neotropic | *Abelmoschus esculentus* | Afrotropic or Indomalaya | NIL | 4 (16, 24, 31, 202) | 6 (7, 42, 68, 134, 182, 295) | NIL | 2 (166, 167) |
| Rosaceae | Nearctic | *Prunus dulcis* | Palearctic | 4 (72, 208, 210, 227) | NIL | 9 (3, 6, 154, 171, 177, 178, 268, 279, 294) | 1 (147) | NIL |
| Rosaceae | Nearctic | *Malus domestica* | Palearctic | 8 (21, 100, 128, 131, 141, 148, 159, 300) | 1 (190) | 8 (43, 75, 76, 192, 240, 241, 290, 299) | 12 (13, 38, 49, 110, 115, 135, 175, 255, 263, 292, 298, 303) | 1 (113) |
| Rosaceae | Nearctic | *Prunus armeniaca* | Palearctic | 1 (317) | NIL | NIL | 1 (292) | NIL |
| Rosaceae | Nearctic | *Prunus persica* | Palearctic | 4 (67, 128, 224, 252) | NIL | 3 (153, 172, 177) | 1 (298) | 1 (188) |
| Rosaceae | Nearctic | *Pyrus communis* | Palearctic | 4 (92, 128, 148, 159) | NIL | 2 (5, 51) | 2 (292, 298) | 1 (113) |
| Rosaceae | Nearctic | *Prunus domestica* | Palearctic | 1 (67) | NIL | 4 (43, 51, 170, 199) | 2 (106, 298) | NIL |
| Rosaceae | Nearctic | *Rubus idaeus* | Palearctic | 5 (73, 200, 233, 234, 306) | NIL | NIL | 3 (78, 164, 174) | 5 (185, 197, 198, 257, 258) |
| Rosaceae | Nearctic | *Fragaria × ananassa* | Nearctic | 4 (17, 39, 146, 265) | NIL | 4 (4, 9, 304, 311) | 4 (63, 115, 204, 225) | 1 (253) |
| Rosaceae | Nearctic | *Prunus avium* | Neotropic | 4 (91, 107, 127, 264) | NIL | 1 (177) | 1 (292) | NIL |
| Solanaceae | Neotropic | *Capsicum annuum* | Neotropic | NIL | NIL | 2 (61, 262) | 1 (307) | 5 (79, 94, 101, 220, 246) |
| Solanaceae | Neotropic | *Capsicum frutescens* | Neotropic | NIL | 1 (23) | NIL | NIL | 1 (206) |
| Solanaceae | Neotropic | *Solanum melongena* | Indomalaya | NIL | 4 (23, 111, 205, 212) | 10 (2, 44, 50, 61, 68, 77, 191, 219, 291, 295) | 1 (161) | 4 (22, 181, 184, 313) |
| Solanaceae | Neotropic | *Solanum lycopersicum* | Neotropic | 2 (283, 293) | 1 (23) | 3 (61, 68, 304) | 5 (54, 116, 236, 248, 307) | 10 (82, 87, 101, 102, 163, 249, 260, 273, 274, 302) |

**Table S4**: crop and family origins, according to the most recently published studies.

| Crop | Family origin | Crop origin |
| --- | --- | --- |
| *Carthamus tinctorius* (safflower) | Asteraceae is thought to have originated in southern South America (Neotropics) (1). | *C. tinctorius* was domesticated in the Near East/West Asia (Palearctic) (2). |
| *Helianthus annuus* (sunflower) |  | *H. annuus* was domesticated in eastern North America (Nearctic) (3). |
| *Citrullus lanatus* (watermelon) | The Cucurbitaceae family originated 70 million ybp in the area we now call Indomalaya (Schaefer et al. 2009). | There is controversy as to whether *C. lanatus* was domesticated in sub-Saharan Africa or in northeast Africa (Palearctic) (4, 5), so we coded it as having an Afrotropical and Palearctic origin. |
| *Cucumis melo* (muskmelon) |  | *C. melo* is thought to have been domesticated in Indomalaya (Sebastian et al. 2010). |
| *Cucumis sativus* (cucumber) |  | *C. sativus* is thought to have been domesticated in Indomalaya (Sebastian et al. 2010). |
| *Cucurbita pepo* (squash) |  | *C. pepo* appears to have been domesticated twice, once in eastern North America (Nearctic) and once somewhere on the Neotropic-Nearctic border in Mexico (6, 7). We coded *C. pepo* as Nearctic and Neotropic. |
| *Cucurbita moschata* (pumpkin) |  | *Cucurbita moschata* was domesticated in the northern Neotropics (Kistler et al. 2015; Kates et al. 2017). |
| *Cajanus cajan* (pigeon pea) | The origin of Fabaceae is less well understood and could have been the Palearctic or Afrotropic (8). | *C. cajan* was domesticated in India (Indomalaya) (9). |
| *Glycine max* (soybean) |  | *G. max* was domesticated in southern China, somewhere near the Palearctic-Indomalaya border (10), and so was coded as originating in both realms (though literature describing bee visitors was only found from the Palearctic). |
| *Phaseolus vulgaris* (green bean) |  | *P. vulgaris* was domesticated independently twice; once in the Andes (Neotropics) and once in Mexico somewhere around the Neotropic-Nearctic border (11) and so was coded as originating in the Neotropics and Nearctic. |
| *Phaseolus coccineus* (runner bean) |  | We could not find recent literature investigating the origins of *P. coccineus* and so assume the origin to be the same as for its congener *P. vulgaris.* |
| *Vigna unguiculata* (black-eyed pea) |  | *V. unguiculata* was domesticated in West or East Africa (Afrotropic) (12). |
| *Gossypium hirsutum* (upland cotton) | Malvaceae is thought to have originated in South America (Neotropics) (13, 14). | *G. hirsutum*, the only cotton for which we could obtain sufficient flower observation data) appears to have originated on the Yucatan peninsula in Mexico (Neotropics) (15). |
| *Abelmoschus esculentus* (okra) |  | The origin of *A. esculentus* may be either Indomalaya or the Afrotropics (16). |
| *Fragaria × ananassa* (the garden strawberry) | Rosaceae appears to have originated in North America (Nearctic) (17). | *Fragaria × ananassa* was first cultivated in Europe as a hybrid of two New World species that also occurs as a natural hybrid in the Nearctic (18, 19), so we coded strawberry as having a Nearctic origin. |
| *Malus domestica* (apple) |  | *M. domestica* originated in Central Asia (Palearctic) and was domesticated along the Palearctic section of the Silk Road (20). |
| *Prunus* (peach, plum, almond, apricot, and cherry) |  | All *Prunus* crops included in this study were domesticated in the Palearctic (21-24). |
| *Pyrus communis* (pear) |  | *P. communis* was also domesticated in the Palearctic (25). |
| *Rubus idaeus* (raspberry) |  | *R. idaeus* was domesticated in the middle east or Europe (Palearctic) (26). |
| *Capsicum frutescens* (Tobasco chilli) | Solanaceae originated in South America (Neotropics) (27). | *C. frutescens* was domesticated in the Neotropics (28). |
| *Capsicum annuum* (bell and chilli pepper) |  | *C. annuum* appears to have been domesticated along the Neotropic-Nearctic border but from a wild progenitor that was confined to the Neotropics (29), so we have coded it as Neotropical. |
| *Solanum lycopersicum* (tomato) |  | It appears that the wild progenitor of *S. lycopersicum* and its early domesticated form originated in the Neotropics, but was transported to Mesoamerica where its domestication was completed (fruit size enlarged) somewhere near the Neotropic-Nearctic border (30). We code tomato as Neotropical because, under this model of origin, its evolutionary history was shared mostly with Neotropical bees. |
| *Solanum melongena* (eggplant) |  | *S melongena* was domesticated in India or southern China (Indomalaya) (31). |

**Table S5**: Bee genera detected visiting crops across more than one biogeographic realm. Black cells indicate the corresponding genus was detected visiting one or more of the 27 crops surveyed in the corresponding realm. White cells indicate the genus was not detected visiting any of the 27 crops surveyed in that realm.

| **Family** | **Genus** | **Neotropic** | **Nearctic** | **Palearctic** | **Afrotropic** | **Indomalay** |
| --- | --- | --- | --- | --- | --- | --- |
| ANDRENIDAE | Andrena |  | z | z |  | z |
|  | Calliopsis | z | z |  |  |  |
|  | Pseudopanurgus | z | z |  |  |  |
|  | Panurginus |  | z | z |  |  |
| APIDAE | Apis | z | z | z | z | z |
|  | Ceratina | z | z | z | z | z |
|  | Eucera | z | z | z | z | z |
|  | Xylocopa | z | z | z | z | z |
|  | Bombus | z | z | z |  | z |
|  | Anthophora |  | z | z | z | z |
|  | Amegilla |  |  | z | z | z |
|  | Thyreus |  |  | z | z | z |
|  | Trigona | z |  |  | z | z |
|  | Nomada |  | z | z |  | z |
|  | Braunsapis |  |  |  | z | z |
|  | Ancyloscelis | z | z |  |  |  |
|  | Centris | z | z |  |  |  |
|  | Diadasia | z | z |  |  |  |
|  | Doeringiella | z | z |  |  |  |
|  | Exomalopsis | z | z |  |  |  |
|  | Florilegus | z | z |  |  |  |
|  | Melissodes | z | z |  |  |  |
|  | Melitoma | z | z |  |  |  |
|  | Ptilothrix | z | z |  |  |  |
|  | Thygater | z | z |  |  |  |
| COLLETIDAE | Colletes | z | z | z | z | z |
|  | Hylaeus | z | z | z |  |  |
| HALICTIDAE | Halictus | z | z | z | z | z |
|  | Lasioglossum | z | z | z | z | z |
|  | Nomia |  | z | z | z | z |
|  | Pseudapis |  |  | z | z | z |
|  | Sphecodes |  | z | z |  | z |
|  | Lipotriches |  |  |  | z | z |
|  | Nomioides |  |  | z |  | z |
|  | Augochlora | z | z |  |  |  |
|  | Augochlorella | z | z |  |  |  |
|  | Augochloropsis | z | z |  |  |  |
|  | Agapostemon | z | z |  |  |  |
| MEGACHILIDAE | Megachile | z | z | z | z | z |
|  | Coelioxys |  | z | z | z | z |
|  | Osmia |  | z | z |  | z |
|  | Anthidium |  | z |  | z |  |
|  | Heriades |  |  | z | z |  |
|  | Lithurgus |  |  | z | z |  |
|  | Pseudoanthidium |  |  | z | z |  |

**Table S6:** Bee genera detected visiting crops in one biogeographic realm only.

|  | **Neotropic** | | **Nearctic** | **Palearctic** | **Afrotropic** | **Indomalay** |
| --- | --- | --- | --- | --- | --- | --- |
| ANDRENIDAE | Acamptopeum | Oxaea | Perdita |  |  |  |
|  | Anthrenoides | Psaenythia |  |  |  |  |
|  | Callonychium |  |  |  |  |  |
| APIDAE | Alepidosceles | Mourella | Epeolus | Tarsalia | Allodape | Geniotrigona |
|  | Arhysoceble | Nannotrigona | Habropoda | Melecta | Dactylurina | Lophotrigona |
|  | Diadasina | Paratrigona | Holcopasites |  | Hypotrigona | Tetragonula |
|  | Epicharis | Partamona | Svastra |  | Macrogalea |  |
|  | Euglossa | Plebeia | Triepeolus |  | Melecta |  |
|  | Eulaema | Scaptotrigona | Xeromelecta |  | Meliponula |  |
|  | Eufriesea | Schwarziana |  |  | Pachymelus |  |
|  | Frieseomellita | Tapinotaspoides |  |  | Plebeina |  |
|  | Geotrigona | Thalestria |  |  |  |  |
|  | Manuelia | Tetragona |  |  |  |  |
|  | Melipona | Tetragonisca |  |  |  |  |
|  | Melissoptila | Tetrapedia |  |  |  |  |
|  | Melitomella | Trigonisca |  |  |  |  |
| COLLETIDAE | Cadeguala |  |  |  |  |  |
|  | Ptiloglossa |  |  |  |  |  |
|  | Sarocolletes |  |  |  |  |  |
| HALICTIDAE | Ceratalictus | Pseudagapostemon | Dieunomia |  | Systropha | Ceyalictus |
|  | Corynura | Pseudaugochlora | Paragapostemon |  | Steganomus | Eupetersia |
|  | Dinagapostemon | Pseudaugochloropsis |  |  | Thrinchostoma | Homalictus |
|  | Neocorynura | Rhinochorynura |  |  | Trinomia | Patellapis |
|  | Paraoxystoglossa | Ruizantheda |  |  |  |  |
|  | Pereirapis |  |  |  |  |  |
| MEGACHILIDAE | Epanthidium | Lithurgus | Ashmeadiella | Anthidiellum | Euaspis |  |
|  |  |  | Dianthidium | Chelostoma | Pachyanthidium |  |
|  |  |  | Hoplitis | Ochreriades |  |  |
|  |  |  | Stelis |  |  |  |
| MELITTIDAE |  |  |  |  | Pseudophilanthus |  |

**Assessments of artefacts and biases that could influence interpretation of results**

*Regression*

Cursory sampling of a crop outside its region of origin would lead to incorrect conclusions if contrasted with thorough sampling in the centre of origin, so we assessed whether crops were more thoroughly sampled in the centre of crop or family origin. Since we treated each crop in each study as an observation in regression modelling, the thoroughness of sampling for each crop is the number of sites and hours over which it was surveyed across all studies in that region. We compared the mean and total number of survey locations and hours across all crops (Table S7 below) and for each crop (Figure S1 below). Across all crops, average and total survey sites and hours were greater when crops were grown outside their regions of origin, providing no evidence of sampling bias leading to inflated crop-visiting bee diversity in the centre of crop origin (Table S7). Average number of sites surveyed was 1.3 times greater in the region of family origin, but total number of sites surveyed was 2 times greater when crops were grown outside the region of their family’s origin (Table S7). Similarly, Figure S1 indicates that only eight of 27 crops (30%) were surveyed at more locations in their realm of origin compared to outside their realm of origin, and these were equally distributed between crops of New and Old World origin (i.e. no evidence of geographic bias in survey effort). Figure S2 indicates that 12 of 27 crops (44%) were surveyed at more sites in their realm of family origin, which also provides no evidence of geographic bias. (We did not produce a similar figure for number of survey hours because only 22% of studies reported survey hours). In conclusion, this assessment suggests no sampling bias leading to incorrect conclusions regarding the effects of crop and family origin.

**Table S7:** showing average and total number of survey sites and survey hours across all crops grown outside their region of origin (away) and in their region of origin (home) and family origin (home). The total number of studies in these categories is also shown (note that total number of studies does not add to 317 as some studies included multiple crops).

|  |  | Crop origin | | Family origin | |
| --- | --- | --- | --- | --- | --- |
|  |  | Away | Home | Away | Home |
| No. survey sites | Average | 4.36 | 3.87 | 3.88 | 4.86 |
|  | Total | 963 | 553 | 1006 | 510 |
| No. survey hours | Average | 50.84 | 47.53 | 48.22 | 53.62 |
|  | Total | 2593 | 1426 | 2893 | 1126 |
| No. studies | Total | 221 | 143 | 259 | 105 |


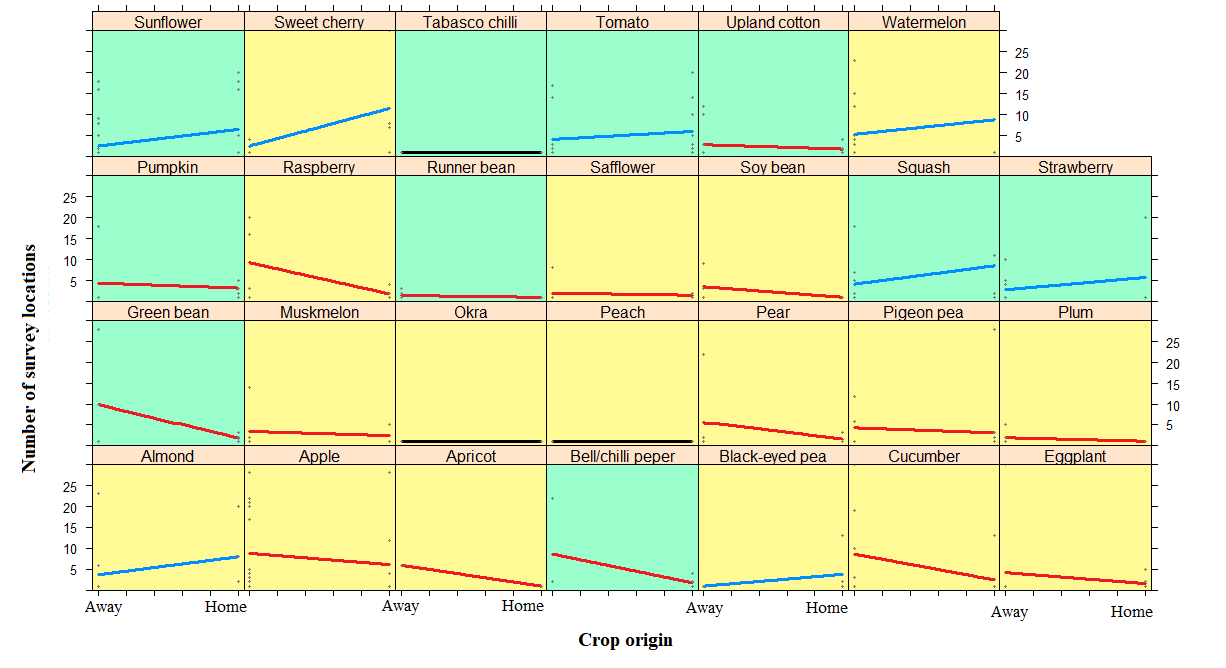


**Figure S1:** Average number of locations surveyed in region(s) of crop origin (Home) compared to region(s) of introduction (Away). Green panel indicates New World crop (Nearctic and/or Neotropic), yellow panel indicates Old World Crop (Palearctic, Indomalaya, and/or Afrotropic). Blue regression line indicates great number of locations surveyed in region of origin, red line indicates greater number of locations surveyed outside region of origin, black line indicates no difference (note that these differences are not necessarily statistically significant).


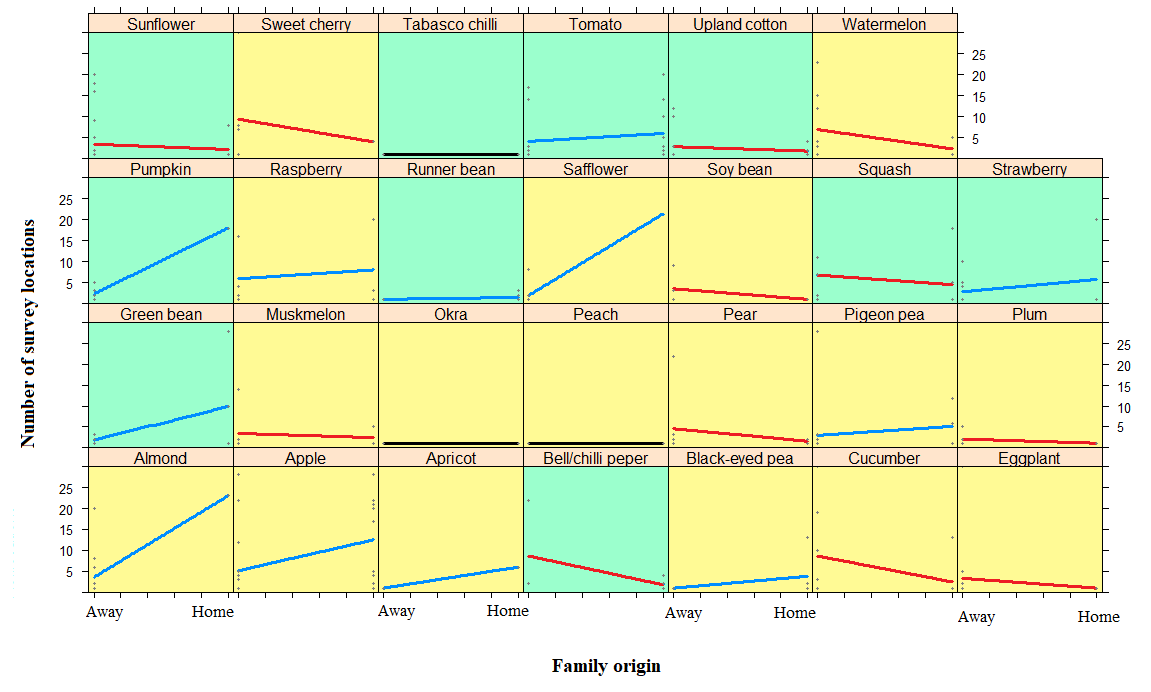


**Figure S2:** Average number of locations surveyed in region(s) of family origin (Home) compared to region(s) outside of family origin (Away). Green panel indicates New World crop (Nearctic and/or Neotropic), yellow panel indicates Old World Crop (Palearctic, Indomalaya, and/or Afrotropic). Blue regression line indicates great number of locations surveyed in region of origin, red line indicates greater number of locations surveyed outside region of origin, black line indicates no difference (note that these differences are not necessarily statistically significant).

Additionally, the effect of crop origin could be an artefact of geographic bias in the origins of crops used in the present study aligning with geographic variation in bee diversity. To demonstrate that effects are reciprocal, i.e. that the same patterns apply for introductions from New to Old World and Old to New world, we have produced Figures S3 below using a regression model identical to the one presented in the paper (i.e. negative binomial model with all the same predictor variables), but with crop species treated as a fixed rather than random effect (i.e. using a GLM rather than GLMM), and an interaction between crop and crop origin (note that these interactions were not statistically significant as 95% confidence intervals overlapped 1). Figure S4 shows the difference in mean number of bee genera observed visiting each crop when grown in its region of origin and outside this region. Twenty of the 27 crops (74%) show some indication of greater crop visitor diversity when grown in the region of origin, with similar numbers of these crops originating in the New World (n = 9) and Old World (n = 11). This shows that effects are reciprocal.


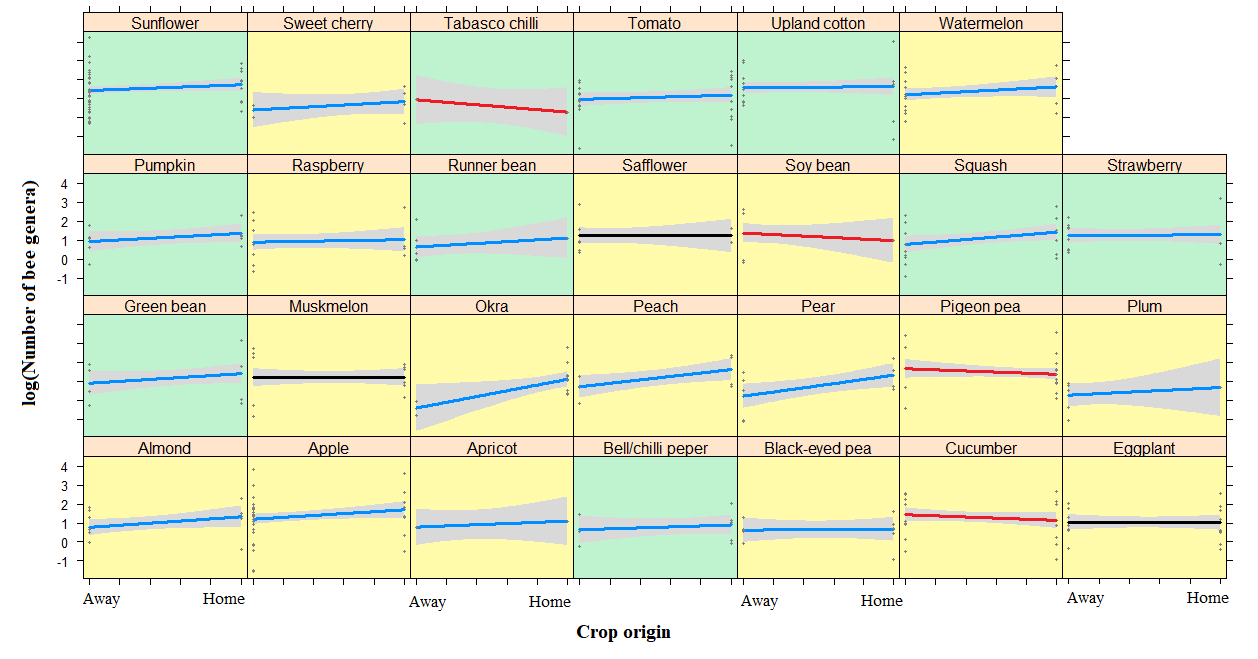


**Figure S3:** Average number of bee genera visiting each crop in region(s) of origin (Home) compared to region(s) of introduction (Away). Green panel indicates New World crop (Nearctic and/or Neotropic), yellow panel indicates Old World Crop (Palearctic, Indomalaya, and/or Afrotropic). Blue regression line indicates great visitation in region of origin, red line indicates greater visitation outside region of origin, black line indicates no difference (note that these differences are not necessarily statistically significant).

To show that the effects are reciprocal using the GLMM we have also tested for an interaction between the effects of crop origin and biogeographic realm. A significant interaction would indicate that effects of crop origin depend on the biogeographic realm where observations were made, which is equivalent to saying that only crops originating in certain realms are visited by fewer bee genera outside their realm of origin (because crop origin for a given crop was 1 when the crop was surveyed in its realm of origin and 0 when it was surveyed in any other realm). The best model used in regression analysis (the full model) was compared by AICc to the same model but with an interaction between crop origin and biogeographic realm. The simpler, additive model (i.e. the one used for inference in the manuscript) was substantially better than the model containing the interaction term (∆ AIC = 6.64) and had 96% of the Akaike weight. Exponentiated 95% confidence intervals for the interaction term overlapped 1 and were therefore not statistically significant at the alpha = 0.05 level).

*Genus accumulation curves*

We also examined the possibility of sampling artefacts in genus accumulation curves. Since closely related bees tend to visit closely related plants (32), bee genus diversity might increase with crop taxonomic diversity, and native crop assemblages might support more bee genera if they are more taxonomically diverse. However, this did not appear to explain the pattern because Shannon diversity (= - ∑ p*_i_* ln p*_i_*, where p*_i_* is the proportion of crop species belonging to the *i*th family) was higher for exotic crops in all but the Neotropic network. Further, when native crop species were removed from the Neotropic network to ensure Shannon diversity was higher for exotic crops, native crops continued to support greater bee genus diversity (95% confidence intervals around native and exotic crop curves did not overlap above five crops, data not shown).

Bee genera accumulated more rapidly with the addition of native compared to exotic crops in the predominantly tropical Neotropic, Afrotropic, and Indomalay, but not the predominantly temperate Nearctic and Palearctic. These differences in bee genus accumulation are unlikely to be sampling artefacts, because the ratios of number of studies for native compared to exotic crops were similar (native:exotic crop studies; temperate = 1.5 and 1.6, tropical = 0.79-1.9), The ratios of number of locations studied for native compared to exotic crops were similar (native:exotic crop locations; temperate = 1.3 and 2, tropical = 1.1-4), and the highest proportions of native crops occurred in the Nearctic and Palearctic realm networks (57% and 60%, compared to 38%-48% in the other realm networks).

Data S1. (<https://doi.org/10.5061/dryad.np5hqbzp5>)

These data include for each observation (i.e. single crop within single publication): the crop species, common, and family name; biogeographic realm and latitude of observation; number of locations surveyed; whether the realm of observation is the crop’s origin (no = 0, yes = 1) or crop’s family origin (no = 0, yes = 1); the number of bee genera observed visiting the crop; and the reference and reference number for the observation (i.e. the publication from which these data were extracted).

**References**

1. Funk V, Randall J, Chan R, Watson L, Gemeinholzer B, Schilling E, et al., editors. Everywhere but Antarctica: Using a supertree to understand the diversity and distribution of the Compositae. Plant Diversity and Complexity Patterns: Local, Regional and Global Dimensions: Proceedings of an International Symposium Held at the Royal Danish Academy of Sciences and Letters in Copenhagen, Denmark,; 2005: Kgl. Danske Videnskabernes Selskab.

2. Chapman MA, Hvala J, Strever J, Burke JM. Population genetic analysis of safflower (Carthamus tinctorius; Asteraceae) reveals a Near Eastern origin and five centers of diversity. American Journal of Botany. 2010;97(5):831-40.

3. Blackman BK, Scascitelli M, Kane NC, Luton HH, Rasmussen DA, Bye RA, et al. Sunflower domestication alleles support single domestication center in eastern North America. Proceedings of the National Academy of Sciences. 2011;108(34):14360-5.

4. Renner SS, Sousa A, Chomicki G. Chromosome numbers, Sudanese wild forms, and classification of the watermelon genus Citrullus, with 50 names allocated to seven biological species. Taxon. 2017;66(6):1393-405.

5. Paris HS. Origin and emergence of the sweet dessert watermelon, Citrullus lanatus. Annals of Botany. 2015;116(2):133-48.

6. Kates HR, Soltis PS, Soltis DE. Evolutionary and domestication history of Cucurbita (pumpkin and squash) species inferred from 44 nuclear loci. Molecular Phylogenetics and Evolution. 2017;111:98-109.

7. Kistler L, Newsom LA, Ryan TM, Clarke AC, Smith BD, Perry GH. Gourds and squashes (Cucurbita spp.) adapted to megafaunal extinction and ecological anachronism through domestication. Proceedings of the National Academy of Sciences. 2015;112(49):15107-12.

8. Doyle JJ, Luckow MA. The rest of the iceberg. Legume diversity and evolution in a phylogenetic context. Plant Physiology. 2003;131(3):900-10.

9. Kassa MT, Penmetsa RV, Carrasquilla-Garcia N, Sarma BK, Datta S, Upadhyaya HD, et al. Genetic patterns of domestication in pigeonpea (Cajanus cajan (L.) Millsp.) and wild Cajanus relatives. PLoS One. 2012;7(6):e39563.

10. Guo J, Wang Y, Song C, Zhou J, Qiu L, Huang H, et al. A single origin and moderate bottleneck during domestication of soybean (Glycine max): implications from microsatellites and nucleotide sequences. Annals of Botany. 2010;106(3):505-14.

11. Bitocchi E, Bellucci E, Giardini A, Rau D, Rodriguez M, Biagetti E, et al. Molecular analysis of the parallel domestication of the common bean (Phaseolus vulgaris) in Mesoamerica and the Andes. New Phytologist. 2013;197(1):300-13.

12. Xiong H, Shi A, Mou B, Qin J, Motes D, Lu W, et al. Genetic diversity and population structure of cowpea (Vigna unguiculata L. Walp). PLoS One. 2016;11(8):e0160941.

13. Carvalho MR, Herrera FA, Jaramillo CA, Wing SL, Callejas R. Paleocene Malvaceae from northern South America and their biogeographical implications. American Journal of Botany. 2011;98(8):1337-55.

14. Baum DA, DeWitt Smith S, Yen A, Alverson WS, Nyffeler R, Whitlock BA, et al. Phylogenetic relationships of Malvatheca (Bombacoideae and Malvoideae; Malvaceae sensu lato) as inferred from plastid DNA sequences. American Journal of Botany. 2004;91(11):1863-71.

15. Wendel JF, Brubaker CL, Seelanan T. The origin and evolution of Gossypium. Physiology of cotton: Springer; 2010. p. 1-18.

16. Werner O, Magdy M, Ros RM. Molecular systematics of Abelmoschus (Malvaceae) and genetic diversity within the cultivated species of this genus based on nuclear ITS and chloroplast rpL16 sequence data. Genetic Resources and Crop Evolution. 2016;63(3):429-45.

17. Chin S-W, Shaw J, Haberle R, Wen J, Potter D. Diversification of almonds, peaches, plums and cherries–molecular systematics and biogeographic history of Prunus (Rosaceae). Molecular Phylogenetics and Evolution. 2014;76:34-48.

18. Liston A, Cronn R, Ashman TL. Fragaria: a genus with deep historical roots and ripe for evolutionary and ecological insights. American journal of botany. 2014;101(10):1686-99.

19. Njuguna W, Liston A, Cronn R, Ashman T-L, Bassil N. Insights into phylogeny, sex function and age of Fragaria based on whole chloroplast genome sequencing. Molecular Phylogenetics and Evolution. 2013;66(1):17-29.

20. Cornille A, Giraud T, Smulders MJ, Roldán-Ruiz I, Gladieux P. The domestication and evolutionary ecology of apples. Trends in Genetics. 2014;30(2):57-65.

21. Horvath A, Balsemin E, Barbot J-C, Christmann H, Manzano G, Reynet P, et al. Phenotypic variability and genetic structure in plum (Prunus domestica L.), cherry plum (P. cerasifera Ehrh.) and sloe (P. spinosa L.). Scientia Horticulturae. 2011;129(2):283-93.

22. Delplancke M, Alvarez N, Benoit L, Espindola A, I Joly H, Neuenschwander S, et al. Evolutionary history of almond tree domestication in the M editerranean basin. Molecular Ecology. 2013;22(4):1092-104.

23. Mariette S, Tavaud M, Arunyawat U, Capdeville G, Millan M, Salin F. Population structure and genetic bottleneck in sweet cherry estimated with SSRs and the gametophytic self-incompatibility locus. BMC Genetics. 2010;11(1):77.

24. Zheng Y, Crawford GW, Chen X. Archaeological evidence for peach (Prunus persica) cultivation and domestication in China. PLoS One. 2014;9(9):e106595.

25. Silva G, Souza TM, Barbieri RL, Costa de Oliveira A. Origin, domestication, and dispersing of pear (Pyrus spp.). Advances in Agriculture. 2014;2014:1-9.

26. Roach FA. Cultivated fruits of Britain: their origin and history. Oxford: Basil Blackwell Publisher Ltd.; 1985.

27. Dupin J, Matzke NJ, Särkinen T, Knapp S, Olmstead RG, Bohs L, et al. Bayesian estimation of the global biogeographical history of the Solanaceae. Journal of Biogeography. 2017;44(4):887-99.

28. Silverman H, Isbell W. Handbook of South American Archaeology. New York: Springer; 2008.

29. Kraft KH, Brown CH, Nabhan GP, Luedeling E, Ruiz JdJL, d’Eeckenbrugge GC, et al. Multiple lines of evidence for the origin of domesticated chili pepper, Capsicum annuum, in Mexico. Proceedings of the National Academy of Sciences. 2014:6165-70.

30. Blanca J, Cañizares J, Cordero L, Pascual L, Diez MJ, Nuez F. Variation revealed by SNP genotyping and morphology provides insight into the origin of the tomato. PLoS One. 2012;7(10):e48198.

31. Meyer RS, Karol KG, Little DP, Nee MH, Litt A. Phylogeographic relationships among Asian eggplants and new perspectives on eggplant domestication. Molecular Phylogenetics and Evolution. 2012;63(3):685-701.

32. Sydenham MAK, Eldegard K, Hegland SJ, Nielsen A, Totland Ø, Fjellheim S, et al. Community level niche overlap and broad scale biogeographic patterns of bee communities are driven by phylogenetic history. Journal of Biogeography. 2018;45(2):461-72.
